# An Overview on SARS-CoV-2 (COVID-19) and Other Human Coronaviruses and Their Detection Capability via Amplification Assay, Chemical Sensing, Biosensing, Immunosensing, and Clinical Assays

**DOI:** 10.1007/s40820-020-00533-y

**Published:** 2020-11-02

**Authors:** Yasin Orooji, Hessamaddin Sohrabi, Nima Hemmat, Fatemeh Oroojalian, Behzad Baradaran, Ahad Mokhtarzadeh, Mohamad Mohaghegh, Hassan Karimi-Maleh

**Affiliations:** 1 College of Materials Science and Engineering, Nanjing Forestry University, Nanjing, 210037 People’s Republic of China; 2 Jiangsu Co-Innovation Center for Efficient Processing and Utilization of Forest Resources, Nanjing Forestry University, Nanjing, 210037 People’s Republic of China; 3 Department of Analytical Chemistry, Faculty of Chemistry, University of Tabriz, Tabriz, 51666-16471 Iran; 4 Immunology Research Center, Tabriz University of Medical Sciences, Tabriz, Iran; 5 Department of Advanced Sciences and Technologies in Medicine, School of Medicine, North Khorasan University of Medical Sciences, Bojnurd, Iran; 6 Department of Nanobiotechnology, School of Biological Sciences, Tarbiat Modares University, Tehran, Iran; 7 Department of Chemical Engineering, Laboratory of Nanotechnology, Quchan University of Technology, Quchan, Islamic Republic of Iran; 8 School of Resources and Environment, University of Electronic Science and Technology of China, Xiyuan Ave, Chengdu, 611731 People’s Republic of China; 9 Department of Chemical Sciences, University of Johannesburg, Doornfontein Campus, PO Box 17011, Johannesburg, 2028 South Africa

**Keywords:** ELISA, qRT-PCR, Sensing assay, Apta assay, Amplification assay

## Abstract

A novel coronavirus of zoonotic origin (SARS-CoV-2) has recently been recognized in patients with acute respiratory disease. COVID-19 causative agent is structurally and genetically similar to SARS and bat SARS-like coronaviruses. The drastic increase in the number of coronavirus and its genome sequence have given us an unprecedented opportunity to perform bioinformatics and genomics analysis on this class of viruses. Clinical tests like PCR and ELISA for rapid detection of this virus are urgently needed for early identification of infected patients. However, these techniques are expensive and not readily available for point-of-care (POC) applications. Currently, lack of any rapid, available, and reliable POC detection method gives rise to the progression of COVID-19 as a horrible global problem. To solve the negative features of clinical investigation, we provide a brief introduction of the general features of coronaviruses and describe various amplification assays, sensing, biosensing, immunosensing, and aptasensing for the determination of various groups of coronaviruses applied as a template for the detection of SARS-CoV-2. All sensing and biosensing techniques developed for the determination of various classes of coronaviruses are useful to recognize the newly immerged coronavirus, i.e., SARS-CoV-2. Also, the introduction of sensing and biosensing methods sheds light on the way of designing a proper screening system to detect the virus at the early stage of infection to tranquilize the speed and vastity of spreading. Among other approaches investigated among molecular approaches and PCR or recognition of viral diseases, LAMP-based methods and LFAs are of great importance for their numerous benefits, which can be helpful to design a universal platform for detection of future emerging pathogenic viruses.

## Highlights

- Various amplification assays and sensing can be applied for the detection of SARS-CoV-2.
- The outputs of biosensors should be presented quantitatively to obtain more accurate and more accessible results.
- Developing smaller size platforms is one approach toward applying such phone apps, as well as utilizing LFA, biosensors, and nanobiosensors detection techniques.

## Graphical abstract

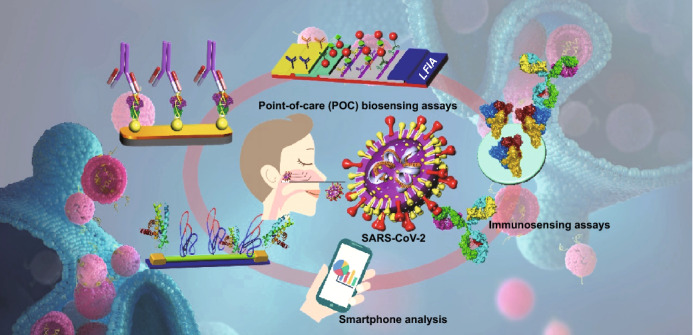

## Introduction

Coronaviruses are known due to their potency to infect human respiratory tracts causing common colds to severe illnesses such as pneumonia. The pathogenesis and epidemiology of these viruses were underestimated for a long time because of the lack of serious threat for human health until the end of 2002 when a novel, unknown severe acute respiratory syndrome (SARS) initiated to involve human, spread all around the world, and cause a high mortality rate. Given the genomic similarity, the SARS coronavirus (SARS-CoV) was introduced to be the causative infectious agent of this disease with more than 8000 cases (lethality rate of 774) [1]. While the mortality rate of this infection and its related disease was not comparable with previous viral pandemics, most of the public concerns about this infection were high global economic costs ($30–100 billion) [2]. Depending on the viral load and the host immune profile, SARS-CoV can involve lower respiratory tracts in fatal pneumonia with the following symptoms: fatigue, headache, muscle pain, loss of appetite, lymphopenia, and rarely diarrhea. Despite many efforts to repress SARS outbreak, the *Coronaviridae* family puts its second pathogenic member forward a decade later, i.e., Middle East Respiratory Syndrome Coronavirus (MERS-CoV) [3].

For the first time, MERS-CoV has been detected in a patient who died from acute respiratory distress syndrome (ARDS) and renal failure in Saudi Arabia during the summer of 2012 [4]. Further studies demonstrated that this new virus could infect camels and bats, as well as humans [5]. The symptoms of MERS-CoV infection were the same as those of SARS-CoV due to its ability to involve lower respiratory tracts; however, the fusion within the host cells in MERS-CoV was mediated by dipeptidyl peptidase-4 (DPP4) receptor differing from angiotensin I, converting enzyme 2 (ACE2) receptors used by SARS-CoV through its entry into the host cells. By 2017, more than 2000 cases of MERS were reported with a mortality rate of approximately 30%. The outbreak of MERS-COV began in Saudi Arabia, but due to air travel, the virus was able to reach other countries in the Middle East, including Jordan, Qatar, Egypt, Kuwait, and the United Arab Emirates, as well as countries outside the region such as Austria, South Korea, the USA, and the UK [6].

As a public health emergency of international concern, the *Coronaviridae* family has been reinforced and emerged with another lower respiratory tract infectious agent, i.e., SARS-CoV-2 and its related disease, Coronavirus Disease 2019 (COVID-19) [7]. The clinical manifestations of this infection were approximately similar to those of SARS, like pneumonia and cough.

**Fig. 1:**
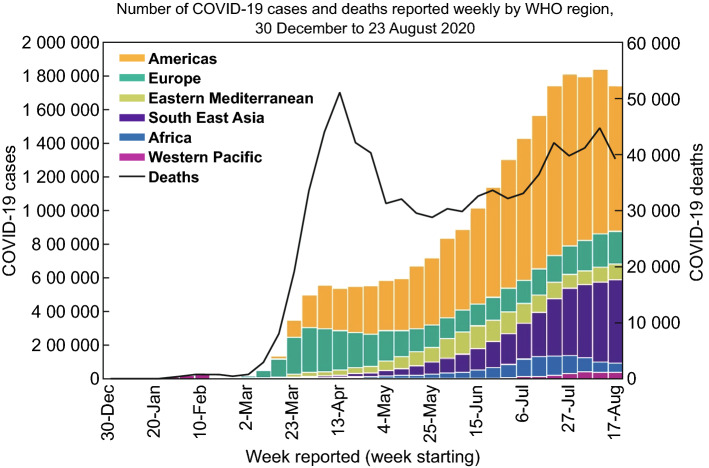
Epidemic chart of confirmed COVID-19 up to August 23, 2020. Used with permission from Ref. [8]. Copyright 2020 World Health Organization

In December 2019, the epicenter of the COVID-19 outbreak was found to be located in Wuhan, China. But, unfortunately, the number of infected countries has increased significantly since the declaration of COVID-19 as a public health emergency of international concern. This virus has spread to more than 130 countries, with over 23.057 M confirmed cases and over 801 K confirmed deaths worldwide as of August 23, 2020 (Fig. 1) [8].

However, the mortality rate of this coronavirus infection seems to be more severe and higher than that of other members. The COVID-19 outbreak is still ongoing and has become a pandemic disease recently [9]. The key strategy dealing with this pandemic is to design a rapid detection sensing system with the following features: paper-based, inexpensive, and available everywhere rather than other detection methods. This strategy can help health care systems to have access to a mass screening at the early stages of infection because the main problem creating this horrible situation is the presence of inappropriate detection systems, which can be feasible after the disease manifestations.

## Coronavirus

**Fig. 2:**
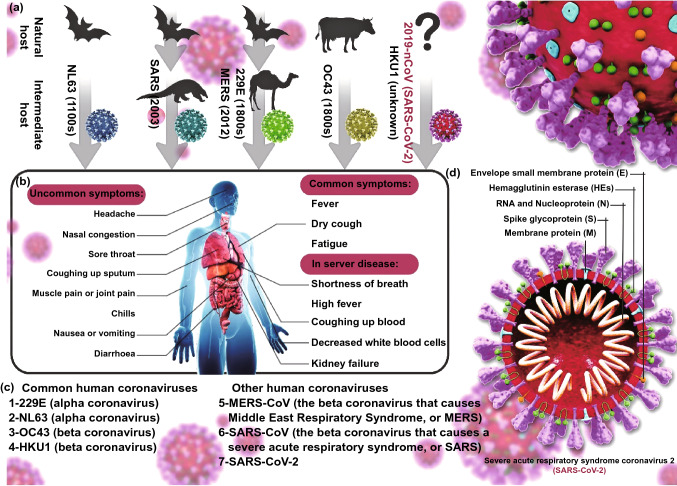
**a** Animal (natural and intermediate hosts) origin of human coronaviruses; **b** clinical presentation of patients with SARS-CoV-2 including common, uncommon, and severe symptoms of SARS-CoV-2. **c** Human coronavirus types: common human coronaviruses; 229E (alpha coronavirus), NL63 (alpha coronavirus), OC43 (beta coronavirus), HKU1 (beta coronavirus), and other human coronaviruses; MERS-CoV (the beta coronavirus that causes Middle East Respiratory Syndrome, or MERS), SARS-CoV (the beta coronavirus that causes the severe acute respiratory syndrome, or SARS), SARS-CoV-2 (the novel coronavirus that causes coronavirus disease 2019, or COVID-19); **d** diagram of coronavirus virion structure showing genome(ranges from 26 to 32 kilobases, the largest for an RNA virus) and structural proteins: spike (S), envelope (E), membrane (M), and nucleocapsid (N). N protein forms a complex with RNA and aids in the viral assembly after its replication; S, E, and M proteins create the viral envelope, and S protein is a club-shaped surface projection, giving the virus its characteristic crown-like appearance on electron microscopy, which is responsible for viral entry into the human cell

*Coronaviridae* is referred to as a family of single-stranded RNA viruses containing 27–32 Kb positive-sense viral genome covered by a bilayer lipidic envelope and a large number of peplomers or spikes on the surface and about 120 nm in diameter. This family is a member of *Nidovirales* order and categorized into 2 subfamilies, 6 genera, 23 subgenera, and about 40 species. Both subfamilies, *Coronavirinae* and *Torovirinae*, can involve humans; however, regarding the scale of involvement and morbidity rate, the genera of *Coronavirinae* subfamily have become more important in comparison with *Torovirinae*’s genera. The *Coronavirinae* subfamily is also classified into 4 genera, *Alphacoronavirus, Betacoronavirus, Gammacoronavirus,* and *Deltacoronavirus*, among which *Alpha*- and *Beta*- are able to infect humans and lead to mild to severe illnesses. SARS-CoV, MERS-CoV, and now SARS-CoV-2 belong to the *Betacoronavirus* genus, having high pathogenic effects on humans, resulting in severe acute lower respiratory tract infections (Fig. 2) [10]. The helical non-segmented RNA of a typical coronavirus encompasses the 5′ cap and 3′ poly(A) tail like a cellular mRNA, which gives rise to the direct translation of viral genome into the functional proteins. Much of the coronavirus genome is made up of its replicase portion (about 20 Kb) that ultimately results in the production of non-structural proteins. The remaining one-third of the genome contains genes that produce the virus’s structural proteins. At the 5′ end of the helical RNA, a leader sequence is located beside the untranslated region (UTR), which includes several stem-loops supporting replication and transcription of the viral genome. Moreover, to control the activity of the structural genes, sequences are embedded at the beginning of these genes, called transcriptional regulatory sequences (TRSs). In general, the formation of a complete coronavirus genome is as follows: 5′-leader-UTR-replicase-Spike (S)-Envelope (E)-Membrane (M)-Nucleocapsid (N)-3′-UTR-poly (A) [11].

**Fig. 3:**
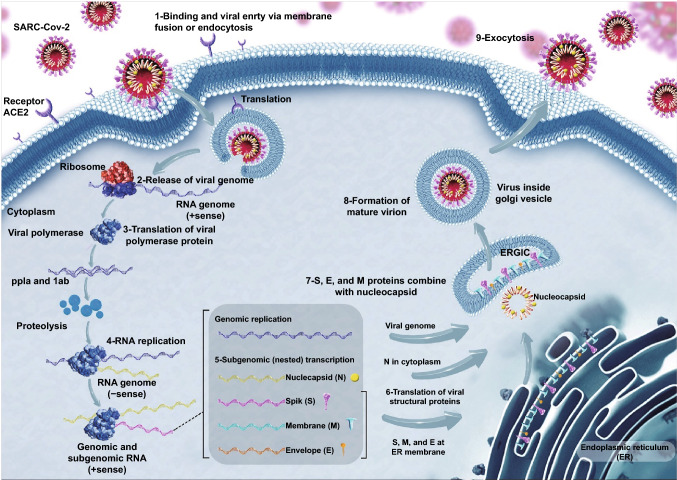
Schematic diagram showing the replication cycle of SARS-CoV-2. The virus moves into the target cells via an endosomal path. Initially, S protein connects to the cellular receptor angiotensin-converting enzyme 2 (ACE2) (1). The viral genome is set free (2) and decoded into viral replicase polyproteins pp1a and 1ab (3); then they are split into small products by viral proteinases (4). Sub-genomic negative-strand templates are created from intermittent transcript on the plus-strand genome and function as patterns for mRNA synthesis (5). The full-length negative-strand pattern is made as a pattern for genomic RNA (6). Viral nucleocapsids are accumulated from genomic RNA and N protein in the cytoplasm (7), followed by budding into the lumen (8), virions are then released from the cell through exocytosis (9)

A complete coronavirus particle is made by the orchestrated formation of S, M, E, and N protein to shape as a spherical and corona solar structure. Once a coronavirus initiates to infect a human cell, the S protein attaches the viral particle to its host cell, facilitating the uncoating process of the virus and triggering the infection [12]. Peptidases are the main protein used as the receptors of coronaviruses independent of their enzymatic function and domain, for example, SARS-CoV and SARS-CoV-2, as well as HCoV-NL63, infect the cells by binding to ACE2 or MERS-CoV while using DPP4 as a way to enter the host cell [6, 13]. The first need for the virus after binding to its receptor is the proteolytic cleavage of the S protein, which is performed by cellular proteases such as cathepsin and TMPRR2 in a pH-dependent manner and results in the fusion of viral and cellular membrane. Following the injection of viral RNA within the cytoplasm, the replicase gene is expressed using the host protein production machine. This gene contains two main ORFs as rep1a and rep1b, which are translated to pp1a and pp1ab polyproteins that subsequently form non-structural proteins (nsps) [14]. Most of these nsps are recruited to shape the replicase–transcriptase complex (RTC) to prepare the proper condition for replication of the viral genome as well as the production of viral structural proteins. Following the replication of viral RNA and synthesizing of structural protein-related mRNA, M, E, and S proteins are produced and transmitted into the endoplasmic reticulum (ER), continuing the protein releasing pathway to make endoplasmic reticulum–Golgi intermediate compartment (ERGIC) [15]. Finally, the N protein encapsidates viral RNA, budding into ERGIC, and the mature virion releases from the cell surface where the cell–cell fusion mediates the viral spread lack of any immunity response and neutralization (Fig. 3) [16].

## Detection Methods

Viral infections are among the main causes of mortality and morbidity in humans. Severe clinical manifestations of COVID-19 have urged scientists to find proper detection methods for the SARS-CoV-2 virus at the early stages of the infection. In contrast to other respiratory viral infections, home hospitalization is not recommended for COVID-19 patients with severe symptoms. Regarding the association between the extent of proinflammatory immune responses and the severity of these symptoms [17], which necessitates timely administration of immune suppressor drugs, late detection of this viral infection can enhance the rate of fatality and impose extra economic costs. Besides, the novel coronavirus, SARS-CoV-2, is highly contagious compared to other viruses, especially respiratory ones. The virus is transmitted through droplets upon sneezing, coughing, and less commonly, close contact exhalation [18]. Asymptomatic carriers of the virus can increase the extent of its transmission. Regarding these unique features of COVID-19, designing specific diagnostic methods for this virus is of particular importance. Early detection of the SARS-CoV-2 virus can hurdle its transmission and help to control the current pandemic. Due to this fact, developing and applying specific and sensitive diagnostic methods for quick detection of the virus is crucial. During the current pandemic and until now, many virus detection techniques have been extensively utilized. These methods include amplifying and sequencing virus-related genes coding, particularly pathogenic proteins [19, 20], detecting the virus in host cells lysates [21], etc. However, these approaches have certain drawbacks that limit their usage as routine point-of-care tests. Cultivation of some viruses cannot proceed fast in cell lines, requiring advanced equipment and specialized workforce, and also high costs are some drawbacks of the above-mentioned techniques. Amplification (i.e., polymerase chain reaction (PCR)-based) assays are highly sensitive, effective, and economically affordable techniques [22–26]. PCR makes it possible to perform complex and real-time investigations. However, the main problems of this outstanding method are the risk of contamination with foreign nucleic acid sources and nonspecific amplification (if improper primers are selected), which hamper with obtained results. In addition, PCR needs skilled personnel and a good laboratory practice. Finally, requiring several hours to be completed makes PCR difficult to be used for emergency bio-recognition purposes.

Next-generation sequencing [27–31] is a highly sensitive and selective technique for revealing new genomic sequences and is considered as an efficient method for clinical purposes. However, demands for this effective method have slightly diminished due to high costs and needs for complicated equipment. Moreover, good laboratory practice is another requirement for this technique. Immunoassay approaches like enzyme-linked immunoassays (ELISAs) which work based on antigen–antibody interactions, are highly sensitive and much quicker than the above-mentioned techniques. However, requiring specific and high-affinity antibodies (and sometimes expensive recombinant antibodies), especially in the case of complex investigations, has limited their application in routine point-of-care procedures. For solving this problem, low-cost analogs of antibodies have gained much attention in experimental studies.

Sensing and biosensing platforms for detecting viruses [32–36] are considered as ideal and outstanding approaches for providing reliable and alternative solutions for real-time diagnostic and continuous monitoring purposes. In recent years, sensing and biosensing assays have attracted great attention as useful and appropriate tools for point-of-care applications because of their rapid response, high sensitivity, low detection limits, portability, and easy fabrication process [37, 38]. Various sensing and biosensing platforms for virus detection have recently been developed. Nowadays, regarding the coronavirus epidemic, it is promising to develop a novel and outstanding biosensing method for early and highly sensitive detection of this fatal virus.

**Fig. 4:**
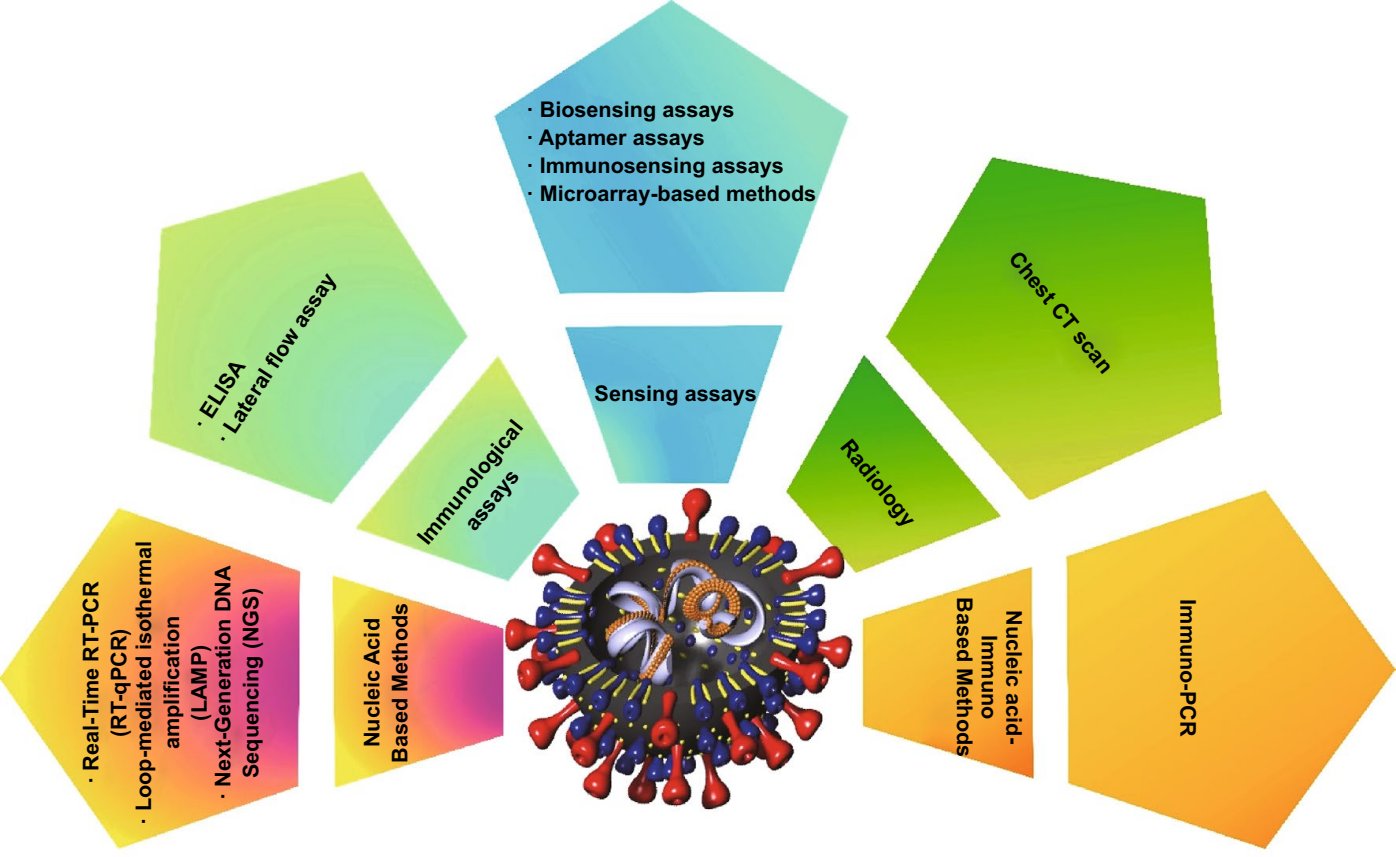
Overview of different developed techniques for the detection of SARS-CoV-2

In this paper, different traditional and novel pathogen sensing methods, along with the methods for detecting human coronaviruses, have been explained in detail. Among the diagnostic methods discussed earlier in this section, ELISA, real-time PCR, loop-mediated isothermal amplification (LAMP), and chest computed tomography (CT) are of great importance for revealing human coronaviruses. On the other hand, various sensing platforms that can efficiently detect human coronaviruses are classified as sensors, biosensors, immunosensors, and aptasensors. The detection mechanism, advantages, and drawbacks of each of these techniques, which are currently used to identify the new coronaviruses (SARS-CoV-2), will be discussed as briefly as possible (Fig. 4).

### Chest Computed Tomography (CT) for Detection of Human Coronaviruses

Chest computed tomography (CT) scan is a kind of radiography and medical imaging using X-ray to figure out any alteration of lung tissue, which sometimes could be helpful to unspecific diagnosis of respiratory viral infections such as coronavirus infection. Regarding the clinical manifestation of COVID-19, CT scan can be used to confirm coronavirus infection as a cause of lung involvement by showing bilateral multilobar ground-glass opacification (GGO) with a peripheral or posterior distribution [30]. A study in Wuhan, China, on COVID-19 patients, showed that CT scans had a sensitivity of about 98%, which is of particular importance compared to a 71% sensitivity of RT-PCR [30]. The findings of CT scan are usually unspecific and are the same as other types of pneumonia; however, the combination of this imaging with other diagnostic techniques such as real-time PCR or biosensing as the confirmation could be useful to recognize COVID-19 patients.

To overcome the technological limitations (i.e., time-consuming, facility requirement, and laborious process) of the traditional methods such as RT-qPCR and ELISA, an alternative technique should be deployed. When end users can access sensing and biosensing devices, it is recommended to modify available technologies in order to continue working with typical systems rather than replacing new devices. The latest coronavirus catastrophe that occurred in China gave us this opportunity to develop new biosensing and sensing devices capable of meeting the demands of inexpensive and user-friendly systems. Considering the great significance of recognizing various coronavirus types, distinctive methods based on sensing and biosensing techniques (such as sensors, biosensors, aptasensors, and immunosensors), designed for the *Coronaviridae* family, have been explored in detail in the following sections.

### Real-Time PCR

Over the past decades, the nucleic acid-based detection of viruses has been used to identify a specific sequence of the viral genome, confirming the viral infection presumed from the symptoms. In comparison with other types of PCR techniques, real-time PCR has some benefits including elevated speed of the process, decreased cycle time, removing of post-PCR procedures such as gel electrophoresis, and decreased amplicon size as well as the quantizing of virus detection instead of the previous YES/NO format [39]. The quantification of the template by PCR can be done in two ways: relative measurement and absolute measurement. The relative quantity explains the variation in the target sequence rate compared to its level in a related matrix. The absolute quantity expresses the exact number of nucleic acid targets in the sample relative to a particular unit [40]. Depending on the type of viral genome (RNA or DNA), the process of nucleic acid isolation and purification could be varied and have some extra steps. In the term of coronavirus infection, several sequences are used to detect the presence of the virus. For example, to indicate the presence of MERS-CoV infection, the upE sequence (upstream of E gene), ORF1b, and ORF1a are amplified by RT-PCR technique [41] or about SARS-CoV, some universal primer pairs are utilized as follows: COR-1/COR-2, BNIoutS/BNoutAs, BNIinS/BNIAs, SAR1s/SAR1as, Cor-p-F, Cor-p-R, and HKU [42]. Recently, following the novel coronavirus (SARS-CoV-2) outbreak, several institutes around the world start to share universal primer pairs, which could find SARS-CoV-2-related genes. These genes contained ORF1ab, N, RNA-dependent RNA polymerase (RdRP), E, ORF1b, and S (Table 1) [43]. Despite the accuracy and specificity of real-time PCR testing, this test is time-consuming and also expensive, as well as the need for expert staff [44].

**Table 1:** Universal primer pairs introduced for the amplification of high pathogenic coronaviruses-related genes [55]

Virus	Sense primer	Anti-sense primer
MERS-CoV	GCAACGCGCGATTCAGTT	GCCTCTACACGGGACCCATA
	TTCGATGTTGAGGGTGCTCAT	TCACACCAGTTGAAAATCCTAATTG
SARS-CoV	CACCGTTTCTACAGGTTAGCTAACGA	AAATGTTTACGCAGGTAAGCGTAAAA
	ATGAATTACCAAGTCAATGGTTAC	CATAACCAGTCGGTACAGCTAC
	GAAGCTATTCGTCACGTTCG	CTGTAGAAAATCCTAGCTGGAG
	CCTCTCTTGTTCTTGCTCGCA	TATAGTGAGCCGCCACACATG
	CTAACATGCTTAGGATAATGG	CAGGTAAGCGTAAAACTCATC
	TACACACCTCAGCGTTG	CACGAACGTGACGAAT
SARS-CoV-2	CCCTGTGGGTTTTACACTTAA	ACGATTGTGCATCAGCTGA
	GGGGAACTTCTCCTGCTAGAAT	CAGACATTTTGCTCTCAAGCTG
	GTGARATGGTCATGTGTGGCGG	CARATGTTAAASACACTATTAGCA
	ACAGGTACGTTAATAGTTAATAGC	ATATTGCAGCAGTACGCACACA
	TGGGGYTTTACRGGTAACCT	AACRCGCTTAACAAAGCACTC
	TAATCAGACAAGGAACTGATTA	CGAAGGTGTGACTTCCATG
	CGTTTGGTGGACCCTCAGAT	CCCCACTGCGTTCTCCATT
	TTCGGATGCTCGAACTGCACC	CTTTACCAGCACGTGCTAGAAGG
	CTCGAACTGCACCTCATGG	CAGAAGTTGTTATCGACATAGC
	TTGGCAAAATTCAAGACTCACTTT	TGTGGTTCATAAAAATTCCTTTGTG
	TCAAGACTCACTTTCTTCCAC	ATTTGAAACAAAGACACCTTCAC
	GACCCCAAAATCAGCGAAAT	TCTGGTTACTGCCAGTTGAATCTG
	TTACAAACATTGGCCGCAAA	GCGCGACATTCCGAAGAA
	GGGAGCCTTGAATACACCAAAA	TGTAGCACGATTGCAGCATTG
	AGATTTGGACCTGCGAGCG	GAGCGGCTGTCTCCACAAGT
	ATGAGCTTAGTCCTGTTG	CTCCCTTTGTTGTGTTGT
	GGTAACTGGTATGATTTCG	CTGGTCAAGGTTAATATAGG
	ACAGGTACGTTAATAGTTAATAGCGT	ATATTGCAGCAGTACGCACACA

Furthermore, detecting of coronavirus RNA in clinical samples needs professional instruments and RNA extraction and purification kit, because the cellular RNAs are also extracted with viral RNAs which could disturb the amplification and detection mechanism. Thus, this test cannot be utilized as rapid testing. Moreover, regarding the instability of RNA genome and also the method of RNA collection and extraction, the chance of false-negative will increase. Also, the RT PCR test cannot tell if a patient has been exposed to the disease and has recovered, or if they are more likely to get the disease. In addition, the coronavirus can mutate its genes, which might disable the primers to detect their specific targets. However, detection of the conserved regions in the viral genome can ameliorate the rate of problems such as detecting the 5′ UTR region, which is well conserved in most of the coronavirus genes. The other important problem with the detection of coronaviruses by real-time PCR is the normalization of expression because the viral genes are transcripted by RdRP instead of cellular RNA polymerase, while using absolute real-time PCR can solve this problem [45].

### Loop-Mediated Isothermal Amplification (LAMP) Assay

To overcome all the technical limitations and laborious detection assays using qRT-PCR, alternative nucleic acid-based method should be deployed. Loop-mediated isothermal amplification (LAMP) is a novel molecular diagnostic technique for the amplification of DNA with high sensitivity and specificity, cost-effectiveness, high efficiency, and rapidity under isothermal conditions, which is 10 times more sensitive than the conventional PCR. Unlike the conventional PCR carried out with a series of repeated temperature changes and 3–40 cycles, LAMP does not require a temperature cycle and is carried out at a constant temperature (60–65 °C). LAMP uses only a set of 4 specific primers and a DNA polymerase enzyme (for example, a large fragment of *Bst* DNA polymerase) with replication activity as well as high strand displacement activity, which amplified target genes up to 10^9^ copies in less than an hour.

The LAMP technique is also performed to detect RNA sequences using reverse transcriptase (RT) together with DNA polymerase called RT-LAMP [46]. The amplified product could be measured by photometry, visualizing the turbidity resulted from the deposition of magnesium pyrophosphate in solution amplified as a by-product [47]. Any changes in the solution can be seen by the naked eyes or by performing very simple photometric techniques using fluorescence dyes such as SYBR green [48]. This novel technique is widely being used as a powerful alternative POC assay for the detection of viral infections. RT-LAMP is a single-stage nucleic acid amplification method that is functionalized to identify infectious disease resulting from viruses or bacteria; it is also a genetic diagnosis approach utilized extensively to detect viruses, requiring just a single temperature for amplification, and is capable of being completed in less than 1 h just inside a dry bath. Nowadays, several viruses are detected using LAMP or RT-LAMP methods such as human influenza A virus (as a RNA virus) [49] and herpes simplex virus (as a DNA virus) [50]. Previously, the RT-LAMP method was used to detect high pathogenic coronaviruses such as SARS-CoV in 11 min at 63 °C as the reaction temperature, contributing to the rapid diagnosis of this infection through 2003 SARS-CoV outbreak [51]. LAMP method can be a potential candidate for the POC device for the detection of new coronavirus, SARS-CoV-2, and its related morbidity, COVID-19. Abundant studies initiate to design a new RT-LAMP protocol for the detection of this new coronavirus by amplifying a single RNA sequence that is unique in the SARS-CoV-2 in comparison with other coronaviruses. The RT-LAMP protocol designed by Park et al. is reported to be able to detect SARS-CoV-2 RNA with at least 100 copies in a sample. They used 69 °C as the reaction temperature followed by 95 °C for 5 min to deactivate RT and melting curve step. However, 5 regions from SARS-CoV-2 were selected to be amplified by this protocol as 2 regions of nsp3, 2 regions of S gene, and 1 region from Orf8; nsp3 region amplification demonstrated more sensitivity than other regions. The other beneficial aspect of this study was low period of reaction (30 min after beginning) [52]. Lamb et al. in the USA defined a new RT-LAMP protocol to detect causative agent of COVID-19, taking much less time up to 30 min and 63 °C for reaction temperature [53]. This new protocol seems to be more affordable, accelerating the detection procedure as well as facilitating the detection, which could make this protocol more reliable than others. The next detection of SARS-CoV-2 was performed by Lin Yu’s team, who designed an RT-LAMP protocol with less detection time up to 15 min [54]. Altogether, the most important benefits of the LAMP technique are its lesser affording and eliminated time-consuming process as well as the need for constant temperature which omits the thermocycler step, the most essential step in the PCR technique. In addition to its advantages, LAMP technique also has some limitation restricting its use, for example, this technique is less used than PCR and is utilized only in clinical identification processes to initiate appropriate treatment time; so, PCR cannot be substituted for biological processes with research purposes such as cloning [55]. Because the LAMP technique uses primer sets (between four and six numbers) and targets several regions of a single piece of DNA or RNA, the design of these primers requires high ability and advanced tools and software that are much more time-consuming and difficult than the PCR technique. Other limitations of LAMP include the need to use primers with degenerated sequences to detect infections, especially with viruses of different types, which can only be feasible by using PCR testing as the diagnostic technique. The large number of primers for each target in the LAMP technique greatly increases the likelihood of primer–primer interactions during this test procedure, which can have a significant effect on the specificity of the test compared to PCR. Another major drawback and limitations of the LAMP technique is the serial presence of DNA products, which causes several bands to emerge after the gel electrophoresis step rather than having a band on the gel, making it difficult to detect each band [56].

### ELISA

**Fig. 5:**
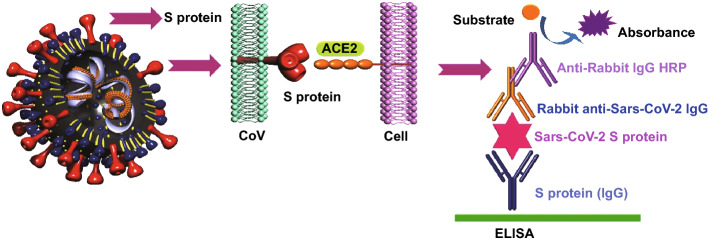
Overview of ELISA techniques for the detection of SARS-CoV-2

For many years, immunological assay, particularly ELISA, is used to detect viral antigens or antibodies against viral antigens in order to assess the rate of viral infection or vaccine efficiency. In fact, the ELISA test is based on the interaction between antigens and the antibodies using an enzyme to visualize and transform the reads in a measurable way. So far, several ELISA methods have been defined based on the material coated at the bottom of plates or the way of measuring the absorbance and exact concentration. These methods are direct ELISA, sandwich ELISA, competitive ELISA, and reverse ELISA [57]. Accomplishing a perfect ELISA test needs at least one antibody specified to detect an antigen which is coated at the bottom of a microtiter plate. Moreover, this test is utilized to measure the amount of specific antibodies in the samples by using a microtiter plate coated with the complement antigen (Fig. 5). Measuring the amount of the desired antigens or antibodies is done by the combination of colorimetric and spectrophotometric in the ELISA test [58].

Given the ability of immune cells to produce antibodies, a person who gets infected with a kind virus initiates to express specific antibodies in his/her circulation, which could be measured by ELISA testing. Depending on the type of antibody, the phase of each viral infection is estimated as, if such antibodies are IgM, they refer to the acute infection and, if they are IgG, they refer to chronic or previous infection [59]. The identification of MERS-CoV from the patient serum using the ELISA technique was feasible by detecting N or S protein-specified antibodies [60]. Moreover, the immune detection of coronaviruses is also utilized to find SARS-CoV and SARS-CoV-2 [61, 62]. Since this test is performed on the patient serum, one of the major problems with the ELISA test is that coronavirus infection is a local type of viral infection and mostly involves the mucosal immunity of the individual.

**Fig. 6:**
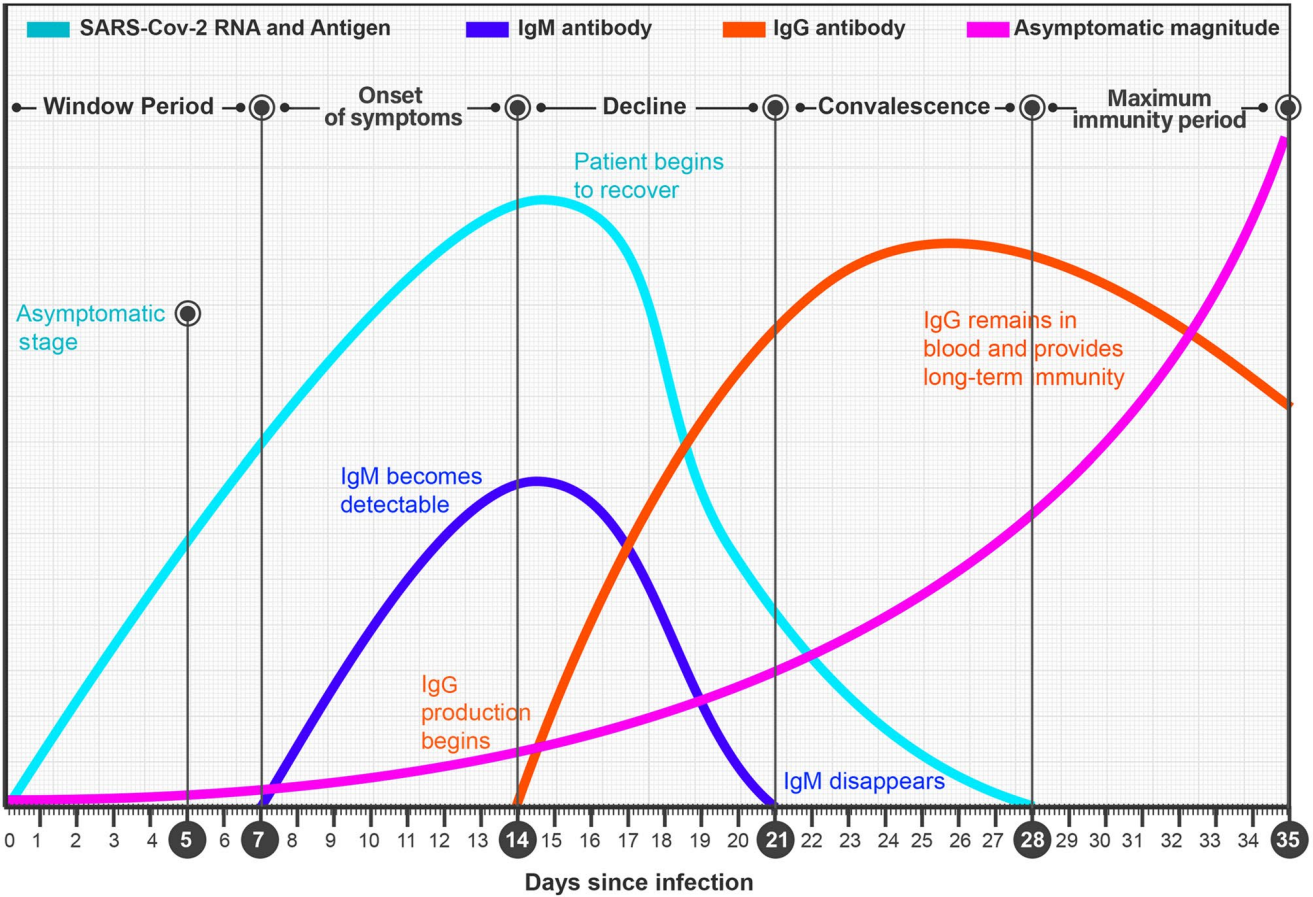
Based on the unsatisfactory efficiency of COVID-19 kits due to the detection lag of 7 and 14 days for appearing IgM and IgG in the serum of asymptomatic patients, porters would not be recognized, triggering the spread [63–66]

Current IgM and IgG kits of coronavirus have intact on coronavirus, which should infect laboratory technicians. Also, the presence of IgM and IgG in the serum of asymptomatic patients is detectable after 7 and 14 days after being exposed to a virus means that the patient would carry a virus while the kit cannot detect it (Fig. 6). Therefore, until the submission of this paper, there is no test for the identification of virus antigens in the serum of patients. In general, there are several problems with this test, which make it inappropriate in some cases. These problems include false negative, noise reaction made by samples, unspecific reaction because of improper plate washing, time-consuming, the difference between reagent concentration in prepared ELISA kits, being expensive, and the need for expert staff with the skill of triggering immunoassay, working with ELISA reader and other related devices, and calculating the exact amount of antigens or antibodies [67, 68]. Taken together, these problems highlight the need for a more efficient alternative approach with a lower sampling rate.

### Sensing Methods

**Fig. 7:**
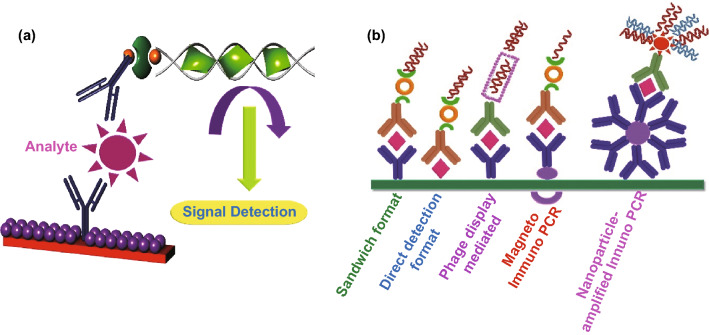
**a** Immune-PCR, the immuno-PCR arrangement begins with a safe assay followed by PCR. Immuno-PCR is parallel to ELISA with the exception that terminal DNA is augmented by a PCR; **b** different immune-PCR platforms available, immuno-PCR, the sandwich setup of immuno-PCR, the direct setup of antigen detection, phage intermediated where single-chain variable fragments (scFv), magneto immuno-PCR, nanoparticle-amplified immune PCR. Redrawn from Ref Front Microbiol. 2019; 10: 1957 [67]

Sensors are outstanding analyzing tools which reversibly and selectively interact with a specific analyte and convert an input measurable chemical parameter to the concentration of a particular compound and an analyzable electrical response [69–73]. The chemical information is produced following the attachment of a chemical compound, biomaterial, or a combination of both to the surface of a physical transducer toward the analyte (Fig. 7).

Up to now, various sensors have been advanced for the detection of diverse kinds of viruses such as human coronavirus [74–77], HIV [78, 79], hepatitis B virus (HBV) [80], hepatitis C virus (HCV) [81], and Zika virus [82, 83].

There has been a growing concern in recent years in the application of sensing systems in the detection of infectious diseases. Microcantilever-based sensors with high sensitivity are currently being developed for the discovery of contagious microorganisms like bacteria, fungi, and viruses. Microcantilevers have emerged as a unique platform for sensors with on-chip electronic circuitry and high sensitivity. The selectivity and specificity of the microcantilever-based sensors can be achieved by the generation of a functional layer on the surface of the microcantilevers by coatings or covalently binding of recognition elements for the detection of targets. In microcantilever-based sensors, cantilever bending induced by differential surface stress is produced when the molecular adsorption is confined to one surface of the cantilever. Microcantilever- based sensor has been used for the detection of SARS-CoV, using feline coronavirus (FIP) type I virus. This microcantilever-based sensor is capable of recognizing (FIP) type I virus with the help of a microcantilever coated by the FIP type I anti-viral serum containing antibodies. The limit of detection was equal to 0.1 μg mL^−1^ for the sensor, and the time of analyzing assay was less than 1 h. Under no circumstances, it would be deduced that the mentioned microsensor is capable of being used to recognize SARS-CoV and FIP I virus in a single and quick test at present, but such outcomes signified that deflecting microcantilevers can sense the coronavirus. This study provides a path to develop the microcantilever sensors for human-related SARS-CoV [84].

**Fig. 8:**
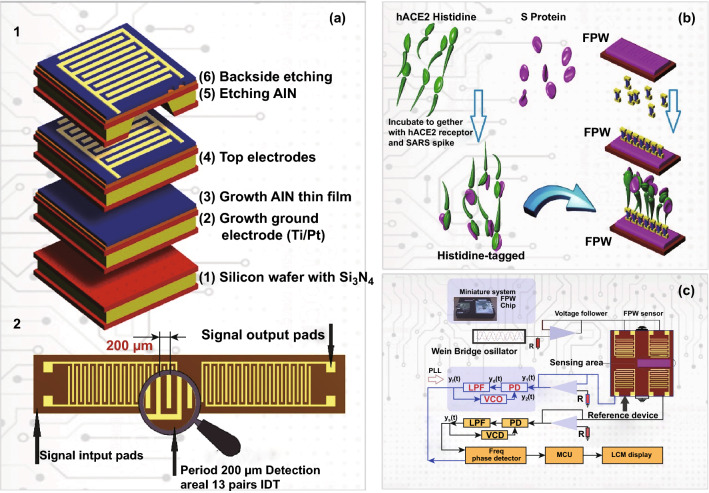
**a** Sensor chip for flexural plate wave. (1) Process of microfabrication with the help of technologies of microelectromechanical systems. (2) Top view image of flexural plate wave where the IDT period is equal to 200 µm along with 13 pairs and 1.6 µm is approximately considered for the total thickness. **b** Diagram of immobilization process represents the fact that an anti-SARS transfer to the sensor system with immobility by the hybrid protein of S-hACE2 can provide phase shifts because of utilizing hACE2 and including the protein S to the functionalized biosensor of flexural plate wave (FPW). **c** Control procedure of the FPW related to the combined minuscule arrangement for sensing and biosensing purposes. The FPW sensing framework system was constructed of a voltage follower, an FPW device, a Wien bridge oscillator (WBO), and a phase-locked loop (PLL) that consist of a phase detector (PD), low-pass filter (LPF) and voltage-controlled oscillator (VCO), a frequency phase detector, a liquid crystal display module (LCM) display, and a microprocessor. Adapted from Ref. (MCU) [86]

The piezoelectric flexural plate wave (FPW) is one of the ultra-sensitive point-of-care promising diagnosis devices with high sensitivity, which can measure the mass of a vibrating element. These microdevices are often created by forming the transducer on the surface of a material or over a substrate with piezoelectric properties [85]. For the detection of SARS-CoV, a potable miniature FPW system developed. Human angiotensin-converting enzyme 2 (hACE2) was employed as a functional receptor for the creation of functionalized FPW biosensor for the detection of SARS S protein. Figure 8 shows the sensor chip for the flexural plate wave-based identification system for SARS coronavirus [86].

**Fig. 9:**
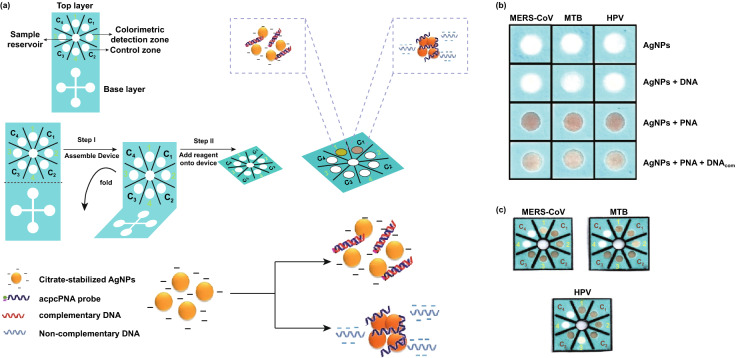
**a** Designing and **b** photograph of visual color alterations got from the recognition of 205 HPV, MTB, and MERS-CoV in the DNAcom presence. After acpcPNA addition, the yellow AgNPs turned red. The color also turned red because of AgNPs aggregation when the solution consists of the DNAnc and acpcPNA. On the other hand, altering the color from red (aggregation state) to yellow (non-aggregation state) in DNAcom presence, the intensity dependence on the DNA concentration is clear. **c** Selecting 100 nM MERS-CoV, MTB, and HPV recognition with the help of a multiplexed colorimetric sensor. Adapted from Ref. [87]. (Color figure online)

Developing the uncomplicated colorimetric and fluorescent assays, which make point-of-care RNA and DNA recognition possible, was the subject of an important study. A colorimetric paper-based assay has been developed for the detection of DNA based on pyrrolidinyl peptide nucleic acid (acpcPNA)-caused nanoparticles aggregation for the screening of MERS-CoV, MTB, and HPV viruses. AgNPs have been utilized as the colorimetric reagent to detect cDNA on the basis of acpcPNA-caused nanoparticle accumulation. Peptide nucleic acid was used as a probe due to its outstanding properties such as high chemically and biologically stable and effective hybridization with complementary DNA strands. The designed acpcPNA probe has a lysine at C-terminal, which gives a positive charge of the probe. In the absence of complementary DNA, the positive charge of the acpcPNA probe leads to aggregation of citrate anion-stabilized silver nanoparticles (AgNPs), while in the presence of the complementary target, DNA and creation of the anionic DNA-acpcPNA duplex lead to electrostatic repulsion. This repulsion causes the dispersion of aggregated AgNPs and, as a result, leads to a detectable color change. The concentration of target oligonucleotide is related to the color change of AgNPs, giving limit of detection 1.53 nM for MERS-CoV. The presented colorimetric DNA sensor based on paper can be used as an alternative method for the selective, sensitive, rapid, and simple assay for MERS-CoV cDNA (Fig. 9) [87].

Nanoparticles present highly sophisticated specifications that allow them to be a part of any novel and promising biological innovation. For example, their size and shape can be modified, and their large surface provides a platform for incorporating various chemical groups as available binding sites [90]. Because of this, it is possible to alter the biological function of these nanomaterials toward the site detecting of target molecules through multiple interactions and actively targeted imaging for sensing and diagnosis of viral infections [88]. The most common metal nanoparticles that have been used to treat viral infection or their detection are silver NPs [89], gold NPs [90, 91], silica and mesoporous [88, 92–94], carbon nanotubes [95, 96], iron oxide NPs [97], etc.

### Biosensing Assays

In general, biosensors [98, 99] are ideal devices for analytical purposes. These valuable tools employ a combination of a specific biological element and a transducer to determine a certain analyte in any biological sample with high sensitivity. Biosensing assays can be categorized according to the types of transducers and bioreceptors. Enzymes [100, 101], nucleic acids (DNA or RNA) [102], antibodies (monoclonal, polyclonal, and recombinant) [103], proteins [104, 105], biomimetics (aptamers and molecularly imprinted polymers (MIPs)) [102, 104, 106], and microbial cells [106] are the main groups of bioreceptors that can be employed to detect various pathogens, especially human coronaviruses. Moreover, another significant part of a biosensor is the transducer, which efficiently transforms the physical and chemical alterations related to the bio-recognition event into quantifiable electrical responses. The transducers can be electrochemical, optical, gravimetric (mass-sensitive), and thermometric (calorimetric) [107, 108]. Currently, electrochemical biosensors are applied for highly sensitive determination of different classes of viruses, including human coronavirus, HIV, HBV, HCV, and dengue virus. These biosensors (i.e., potentiometric [109], amperometric [110–112], and conductometric [113]) play significant roles in various biosensensing fields because of their facile fabrication, small size, facile interpret results, robustness, and low detection and quantification limits. In potentiometric biosensors, a bioreceptor and a transducer are combined for detecting changes in the concentration of ions, and consequently, in these biosensors, the obtained analytical response reflects the concentration of the target analyte. Amperometric bio-recognition assays record current alterations at a particular potential during a constant time. The changes of the current are directly associated with the concentration of the target analyte. Conductometric or impedimetric biosensors are considered as effective electrochemical biosensing platforms in which the alterations in the conductivity of a solution are recorded. Another type of biosensors that are commonly utilized for detecting viruses, especially human coronaviruses, includes optical biosensors. They are the highly sensitive, selective, and rapid and have low limits of detection (LODs) and quantification (LOQs). Furthermore, they allow for real-time monitoring of the measurement process. The mechanism of these interesting biosensors is based on the principle of sensing alterations with the dimensions of photons or light. Various kinds of optical biosensors that have been investigated for different viruses, especially human coronaviruses, are plasmon resonance (SPR) [114], ellipsometric and reflectometric interference spectroscopy (RIfS) [54, 115], colorimetric [54, 116], fluorescent [117], and surface-enhanced Raman scattering (SERS) [118, 119]. Among these optical biosensing assays, SPR biosensors are of great importance as they can directly measure the alterations happening in the refractive index of light on the sensor surface, reflecting the analyte concentration. In the following paragraphs, various types of the above-mentioned electrochemical and optical biosensors used for highly sensitive determination of human coronaviruses are discussed.

For the improvement in the sensitiveness of traditional immunoassay tools and rapid and accurate detection of SARS-CoV in the early stage of infection, a sandwich localized surface plasmon coupled fluorescence (LSPCF) fiber-optic biosensor was applied for the detection of SARS N protein. It has been shown that this protein expressed in the early stage of infection could be detected only 1 day after infection; therefore, it could have diagnostic value and disease monitoring. The LSPCF is excited by localized surface plasmon, where the evanescent field is applied near the core surface of the optical fiber. At the same time, the detection of the fluorescence signal is performed by a photomultiplier tube located beside the optical fiber with high collection efficiency. The LSPCF fiber-optic biosensor demonstrates a capacity for recognizing especially small concentration (~ 1 pg mL^−1^) of SARS-CoV N protein in serum [120].

An SPR (surface plasmon resonance)-based biosensor as a real-time and label-free detection system was introduced for rapid and high-throughput detection of SARS coronavirus. The SPR biosensor was devised for the detection of a respiratory virus-specific oligonucleotide in an SPR biochip. To improve the sensitivity of the developed biosensor, PCR primer was labeled by biotin and utilized to increase the signal by introducing streptavidin following hybridization. Throat swab samples infected by nine common respiratory, including SARS, were tested by the inventive SPR-based biosensor to estimate the specificity, duplicability, and sensitivity of this technique. The findings suggest that the use of a high-throughput gene biochip combined with the SPR technique has the capability to be applied for the effective and swift identification of SARS-CoV among nine common respiratory usual viruses [121].

**Fig. 10:**
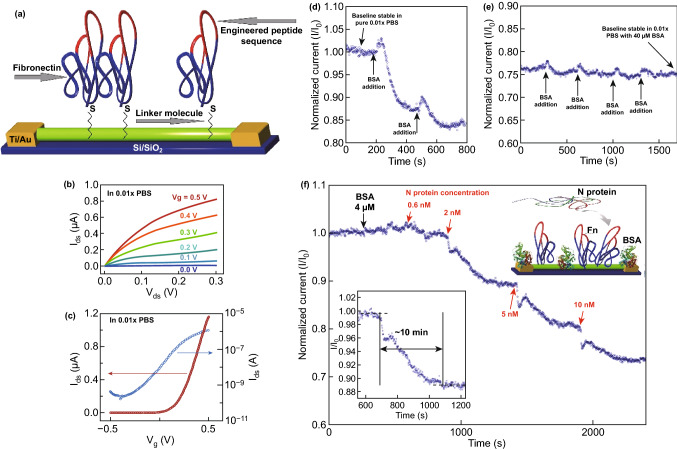
**a** Schematic presentation of immobilized Fn on the exterior part of an In_2_O_3_ nanowire FET apparatus device. The areas of Fn are highlighted in red with the plotted peptide sequence. Fn was connected to the related nanowires through the cysteine sulfhydryl group near the C-terminus, distant from the binding location. **b** Curves pertinent to a family of *I*_ds_–*V*_ds_ and **c** a typical *I*_ds_–*V*_g_ curve (plotted both in logarithmic (blue)) and linear (red) achieved from one of our instruments functioning with the aqueous gate arrangement. Normalized electrical output (*I*/*I*_0_) versus time of a single functioning instrument. **d**–**e** Demonstrating the response curves to passivation upon the addition of consecutive aliquots of BSA. Upon increasing the BSA concentration (from pure 0.01* PBS), the baseline re-equilibrates at lower values of S-D current until stability is finally reached at 40 µM BSA, in 0.01* PBS. **f** Showing response for a nanowire device utilized with Fn. The red arrows show the times when the solution was increased to a specified concentration of N protein. The inset on the right side is the arrangement of our device through active sensing measurements. BSA protein was used to block sites for nonspecific binding. The Fn probe molecule was then used to specifically capture the target N protein. The inset on the left side is to show the plateau and the definition of response time. Adapted from Ref. [122]. (Color figure online)

Detection of specific antibodies, including IgM and IgG of SARS in the blood of infected patients, could be used for highly sensitive, fast, and simple diagnosis methods for the rapid detection of this virus. AMPs (antibody mimic proteins) are polypeptides that attach to their target analytes with great attraction, selectivity, and particularity, just similar to typical antibodies; however, their size is smaller than 2–5 nm and less than 10 kDa, and in contrast with usual antibodies, they show high stability to electrolyte concentrations and a wide range of pH. Nanowire biosensors based on AMP (fibronectin, Fn) as a capture agent with a high binding affinity for nucleocapsid (N) protein have been introduced for the detection of SARS-CoV. This protein is highly antigenic and might be a suitable diagnostic biomarker. The results of this study show that N protein could be detected at sub-nanomolar concentration short response time (~ 10 min) and without any labeled reagents as a signal amplifier when compared to the long time (~ hours) required to achieve a result from other diagnostic technologies such as qRT-PCR and ELISA methods and without any required multistep analysis. This report shows the capability of fabricated nanobiosensors for being used as a precise, suitable, and rapid means for detection of N protein as SARS-CoV infection biomarker (Fig. 10) [122].

**Fig. 11:**
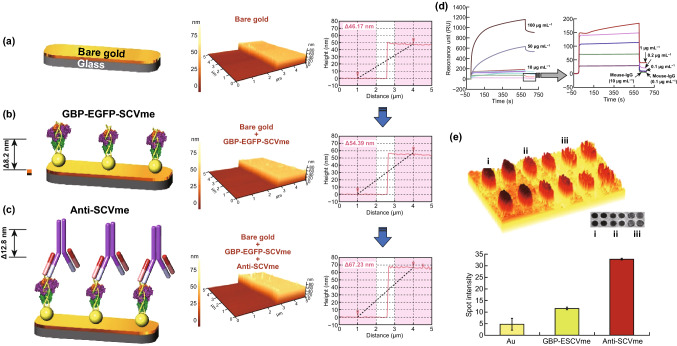
AFM pictures of the consecutive binding of anti-SCVme on the gold-micropatterned surface and GBP-E-SCVme. **a** Bare Au surface, **b** immobilizing of the GBP-E-SCVme fusion proteins onto the gold surface, and **c** consequent connection of the anti-SCVme antibodies on the GBP-E-SCVme layer. Left, schematic of consecutive binding of GBP-E-SCVme and anti-SCVme on the gold micropatterns; middle, three-dimensional topological images; right, the cross-sectional contours of samples **a**–**c**, sequentially (these are average height differences of the individual scan lines from each area). **d** SPR sensorgrams for (1) sensitive and (2) selective recognition of anti-SCVme utilizing the GBP-E-SCVme-imbedded gold sensor chip at different concentrations (0.1, 1, 10, 50, and 100 µg mL^−1^) of anti-SCVme and (1 and 10 µg mL^−1^) of mouse IgG as negative controls. SPRi analysis of the sequential binding of GBP-E-SCVme and anti-SCVme onto gold micropatterns composed of 50-nm-diameter circles. **e** Three-dimensional and two-dimensional (inset) images of bare gold micropatterns as controls (sample (i)); binding of GBP-E-SCVme fusion proteins onto the gold patterns (sample (ii)); and successive binding of GBP-E-SCVme and anti-SCVme onto the gold patterns (sample (iii)). Spot intensities of the three samples shown in scanned images were measured through the gold circle micropatterns. Adapted from Ref. [123]

Another SPR-based biosensor was developed for easy detection of SARS employing a recombinant protein made by genetically fusion gold binding polypeptides (GBPs) to a SARS coronaviral surface antigen (SCVme). The fusion protein offers an easy and actual method for the fabrication of SPR sensing platforms, allowing precise and careful detection of the anti-SCVme antibody. In this fabrication, the GBP domain with high gold binding affinity serves as an anchoring part onto the gold surface, and the SCVme domain acts as a recognition ligand for the detection of anti-SCVme antibodies. SPR analysis indicated that fusion protein self-immobilized onto the gold surface simply and strongly, via GBP, without complicated surface chemical modification, offering a particular sensing platform with high stability for anti-SCVme diagnosis. The desired packing density of the fusion protein to the SPR chip was realized at the concentration of 10 µg mL^−1^; this density presented the best diagnosis response (906 RU) for anti-SCVme. The fusion protein-coated SPR chip at the most favorite packing density had a lower LOD of 200 ng mL^−1^ anti-SCVme in 10 min with high selectivity detection in the presence of nonspecific mouse IgG as the negative control (Fig. 11) [123].

Today, biosensors can be prepared and modified using nanotechnology and applying a range of nanomaterials to improve the specificity and sensitivity of the detection of biomolecules. This is feasible because nanomaterials can upgrade optical and electrochemical functions of biosensors. Furthermore, the detecting capability, selectivity, efficiency, and specificity of biosensors can be improved by designing and incorporating immobilized bioreceptors, which also deliver a more potent signal amplification process, especially when detecting viral particles. By these modifications, we can expect that biosensor-based instruments to soon be used as portable (as they can be very smaller in size than currently available equipment) and affordable (implementing less, cheap, and novel materials) devices at clinical setting [88]. Overall, there is still room for improving the applicability of biosensors for detecting human viruses, in particular coronaviruses, warranting more studies in this field [124].

The biosensor-based method upon imaging ellipsometry was used directly to identify two neutralizing monoclonal antibodies and serial serum samples. Ellipsometry is an optical method for determining the dielectric characteristics (dielectric function or complex refractive index) of thin films. Ellipsometry is based on measuring the alteration of polarization upon transmission or reflection, comparing it to a model. It can be used to characterize thickness (depth), roughness, composition, doping concentration, electrical conductivity, crystalline nature, and other material features. It is very sensitive to the alteration in the optical response of radiation that interacts with the material being examined. As a label-free technique, the biosensor based upon imaging ellipsometry showed a more proficient tool for measuring serum samples from SARS patients and the affinity between these antibodies and the SARS coronavirus [125].

### Immunosensing Assays

Immunoassays are bio-analytical approaches in which the interaction of an analyte (i.e., antigen) and an antibody is the basis of the measuring of a specific analyte [126, 127]. In this regard, an immunosensing assay is designated as an analytical method in which antibodies or antibody parts are used to recognize biomolecules. These assays are frequently applied in different industrial fields such as pharmaceutical, agriculture, and food industries, as well as for biological threat management, epidemic diseases control, and clinical and diagnostics purposes. The produced signal in immunosensing assay is directly related to the rate of antigen–antibody binding events [128–130]. In this regard, the label applied in the immunoassay sensing process should have numerous characteristics: chemical stability, low cost, negligible effect on the binding performance, feasibility, safety, and applicable instrumentation. It is important to say that immunosensing assays are commonly used for high sensitivity detecting of different kinds of viruses, for example, SARS CoV-2 [131–135], HIV [136–138], HBV [139], and HCV [140, 141].

**Fig. 12:**
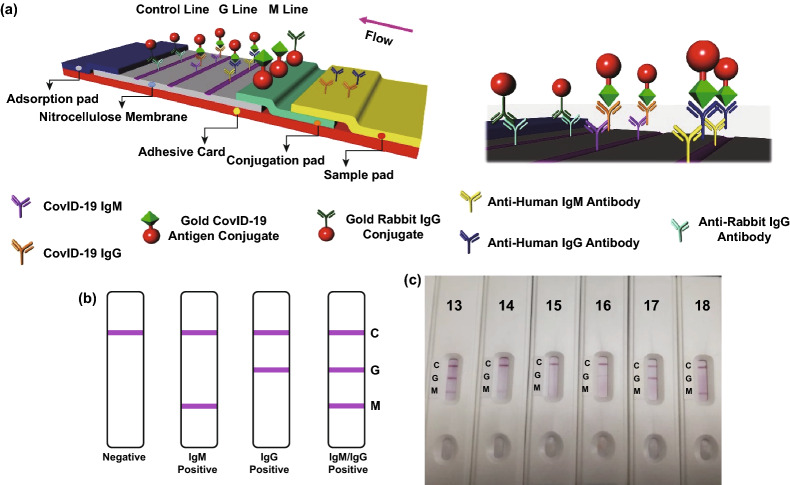
Schematic explanation of quick SARS-CoV-2 IgM–IgG combined antibody test. **a** Schematic of the recognition device. **b** An explanation of various experimental consequences. **c** Relates to the control line, G means IgG line, M pertinent to IgM line. Illustrative photograph for various patient blood experimental consequences. #13 shows both IgG and IgM positive, #14 shows IgM weak positive, #15 illustrate both IgG and IgM negative, #16) IgG weak positive, #17) IgG positive, #18) IgM positive. (Redrawn from Ref. [133])

The incident of the novel coronavirus infection (SARS-CoV-2) immediately spread everywhere throughout the world, and due to severe contagiosum of this virus, it is critical to develop point-of-care (POC) diagnosis assays for monitoring and management of disease in the afflicted area, even though the infection (SARS-Cov-2) nucleic acid RT-PCR test has become the standard strategy for recognition of SARS-CoV-2 disease. Anyway, as a result of these real-time PCR test packs, confinements, and high false-negative rates, there is a pressing need for an exact and rapid diagnostic assay. POCT include tests that analyze patient specimens in place, outside the clinical laboratory. POCTs are often performed by clinical staff without laboratory training or by patients themselves for self-monitoring for obtaining a quick result close to the patient’s bedside. POCT assay based on lateral flow immunoassays (LFIA) is developed for COVID-19 detection and some of the marketed products. Recently, a rapid and portable detection device based on lateral flow immunoassay has been introduced by Liu et al., which can identify SARS-CoV-2 IgM and IgG antibodies at the same time in the blood of infected patients in 15 min which can distinguish patients at various disease stages. Lateral flow tests [142], otherwise called lateral flow immunochromatographic assessments, are straightforward and simple paper-based devices which are gadget-planned to recognize the presence of an objective analyte in a fluid sample without requiring any specific and exorbitant hardware. Lateral flow assays depend on a progression of capillary beds, for example, bits of permeable paper [143], microstructured polymer [144, 145], or sintered polymer. Each of these pads has the ability to spontaneously migrate liquid samples (e.g., saliva, blood, urine). It has been shown that IgM and IgG antibodies can be detected in the blood of patients 3 to 6 days and 8 days after the SARS-CoV infection, respectively. Therefore, recognition of both IgM and IgG antibodies could offer evidence on virus infection time, which could be very helpful for diagnosis and providing effective and timely remedies for COVID-19 patients. The sensitivity, reliability, and specificity of the prepared kit were approved by blood samples provided by 397 qRT-PCR-confirmed COVID-19 patients and 128 negative patients from eight clinical centers. The general testing sensitivity was 88.66% and specificity was 90.63%. Likewise, estimating clinical determination results acquired from various sorts of venous and fingerstick blood tests have been performed. The IgM–IgG combined assay has better utility and sensitivity in comparison with a single IgM or IgG assay. It very well may be utilized for the rapid screening of SARS-CoV-2 carriers, asymptomatic or symptomatic peoples, and is very useful for hospitals, clinics, and laboratories. Furthermore, it can be employed for the detection of infected peoples in businesses, universities, airports, train stations, etc. Figure 12 demonstrates the schematic illustration of rapid SARS-CoV-2 IgM–IgG combined antibody test [133].

**Fig. 13:**
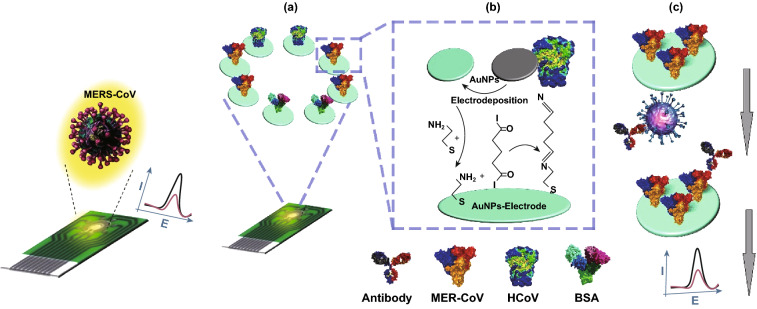
Scheme demonstrating the MERS-CoV immunosensor preparation and also the recognition procedure. The biosensor comprises a competitive immunoassay carried out on DEP array electrodes nanostructured with gold nanoparticles to allow the multiplexed recognition of various CoV. **a** Immunosensor array chip for coronavirus. **b** Stages of immunosensor construction. **c** Recognition procedure of competitive immunosensor for the virus. Redrawn from Ref. [146]

Electrochemical immunosensors have been explored as an appealing choice due to their excellent sensitivity, relatively low cost, ease of use, short response time, and the possibility of miniaturization. Recently, a novel indirect competitive assay based on electrochemical immunosensor has been introduced for the detection of the MERS-CoV virus. The biosensor is based on the indirect competition between the immobilized MERS-CoV protein and free virus in the sample for the fixed amount of added antibodies to the sample (Fig. 13), which was performed on a carbon electrodes (DEP) array changed by the gold NPs. This immunosensor is developed on DPE array to enable the simultaneous and rapid detection of various types of CoV virus. Recombined spike protein S1 has been utilized as a biomarker for MERS CoV. Square wave voltammetry (SWV) has been used to record the electrochemical measurements with the help of ferricyanide/ferrocyanide as a probe. A good linear response from 0.01 to 10,000 ng mL^−1^ and 0.001 to 100 ng mL^−1^ has been monitored for HCoV and MERS-CoV, respectively. The assay time has been performed in 20 min, and the detection limit was as low as 1.0 and 0.4 pg mL^−1^ for MERS-CoV and HCoV, respectively. The prepared disposable DEP array electrode has been promising to lower the cost and enable the multiplex-bead assays for the detection of HCoV and MERS-CoV simultaneously [146].

A piezoelectric (PZ) sensor is an efficient device that uses the piezoelectric effect to measure variations in pressure, temperature, acceleration, force, or strain and convert these changes into an electrical signal. A piezoelectric immunosensor has also been presented to detect SARS-CoV in the sputum in the gas phase. For the preparation of this biosensor, PZ polyclonal antibodies against SARS-CoV were attached to the surface of PZ crystal in an oriented state via protein A. On the other hand, the antigen sample has been atomized into the aerosol with the help of an ultrasonicator so that the antibodies existed on the surface of PZ crystal were capable of specifically adsorbing the SARS-CoV antigen and leading to a change on the crystal mass which could provide a change in the frequency. In optimum situations, changes in frequency depended on the concentration of antigen to an extent from 0.6 to 4 µg mL^−1^ in a linear manner. The device contains short analyzing time (less than 2 min), feasibility, specificity, simplicity, stability (immunosensor has been stable for more than 2 months when stored at the temperature of 4-6 °C over silica gel blue), and great duplicability (it was capable of being reused 100 times without any sensible changes in the activity [147]).

### Apta Assays

Aptamers, which are categorized as a sensing method, are short-chain oligonucleotides (either RNA or single-stranded DNA) that were concurrently introduced by three groups of scientists: Robertson and Joyce, Tuerk and Gold, and Ellington and Szostak [148]. One of the main properties that renders apta-assays as unique is their extraordinary affinity, which is an outcome of their flexibility and the ability to fold upon binding to a target. Their extraordinary advantages are small size, high specificity for target molecules, applicability both in vitro and in vivo, biocompatibility, low costs of production, high molecular stability, and low detection limits (as low as zmol L^−1^). Aptamers, as biological ligands, strongly and selectively bind to the target analyte. Another interesting feature of aptamers is that they effectively preserve their properties in various experimental conditions [149, 150]. Aptasensing bio-assays have various recognition mechanisms and can bind to a wide range of regulatory proteins [138, 151], enzymes [152, 153], mono- and polyclonal antibodies [154], amino acids [155], growth factors [156], toxins [157], low molecular weight vitamins [158], cancer biomarkers [159–161], and even some metal ions [162]. Large quantities of aptamers can be biochemically synthesized most frequently through the systematic evolution of ligands by exponential enrichment (SELEX). Other synthesizing methods include non-equilibrium capillary electrophoresis of equilibrium mixture (NECEEM) [163] and high-throughput aptamer identification screen (HAPIscreen). Aptamers, despite their advantages and versatility, also have disadvantages that may limit their usefulness; these limitations are sensitivity to nuclease degradation, not binding to some targets that lack functional groups, and stronger (and sensitive to enzymatic digestion) bonds with antibodies than target analytes. In the following section, main aptasensing assays for effective determination of human coronaviruses will be discussed.

SELEX referred to as in vitro evolution or in vitro selection is a combinatorial chemistry method in molecular biology for the screening of single-stranded DNA or RNA that specifically binds to a target ligand or ligands with high affinity [164, 165]. Because of the benefits of better stability, simple modification, and easy preparation, aptamers were utilized to fabricate biosensors for detecting infectious microorganisms. The present research work provides the most recent improvements in SELEX to screen aptamers in infectious microorganisms and represents several described aptamers in infective microorganisms (bacteria, viruses, protozoa) and examines biosensors based on aptamer to detect infectious microorganisms. Consequently, the novel movements in biosensors based on aptamers for detecting infectious microorganisms can be debated [166].

SARS-CoV is the etiology-pertained factor for the recently appeared SARS disease. The nucleocapsid protein (N) of SARS-CoV is the most plentiful structure-pertained protein and has the function of a recognition marker for detecting the virus accurately and sensitively. An RNA aptamer with high affinity is selected, which could bind to N protein with a dissociation constant equal to 1.65 nM. Results demonstrated that the selected aptamer can be identified in the C domain of N protein with high specificity in a selective manner. Isolated aptamers can act as a capturing agent for the N protein molecules for the fabrication of an aptamer-based chemiluminescence immunosorbent assay and in a nanoarray aptamer. The prepared aptamer–antibody hybrid immunoassays can detect the low level of N protein (2 pg mL^−1^) with high sensitivity and selectivity. Such aptamer–antibody combined immunoassays can be practically used for the rapid detection of the N protein of SARS-CoV with high sensitivity [167].

Among different SARS-CoV structure-pertained proteins, the protein of nucleocapsid was considered as a better recognition biomarker. An aptamer of ssDNA was isolated against the N protein from a DNA library containing 45 nt with random sequences. The analysis of ELISA shows that this aptamer can identify SARS-CoV nucleocapsid protein with a high binding affinity (with a Kd of 4.93 ± 0.30 nM). Furthermore, the result of western blot analysis confirmed that such an aptamer of ssDNA could effectively detect the SARS-CoV N protein when compared to the nucleocapsid antibodies. Thus, the isolated aptamer of ssDNA can be useful as an alternative detection probe for the rapid and sensitive diagnosis of SARS [168].

The N protein is one of the most important antigens used for the early detection of SARS-CoV infection. An optical biosensor based on quantum dots (QDs)-conjugated RNA aptamer platform with high sensitivity and specificity was designed for rapid diagnosis of SARS-CoV N protein on a chip system. QDs are colloidal nanomaterials belonging to semiconductor materials, which have attracted much attention in the fields of nanomedicine, especially in imaging systems due to their unique optical properties compared with traditional fluorophores in terms of being longer fluorescence lifetime, high stability, and tunable emission spectra [124, 169]. For this purpose, SARS-CoV N protein immobilized on the surface of a glass chip could be effectively hybridized by QDs-conjugated RNA aptamer for the creation of fluorescence signals. The intensity of fluorescence signals is related to the concentration of SARS N protein. This miniaturization device based on optical QDs-RNA aptamer chip can detect SARS-CoV N protein at the concentrations of as low as 0.1 pg mL^−1^. The suggested graphical SARS-CoV N protein method enjoys high sensitivity, accuracy, simplicity, and easy operation [170]. Table 2 presents a comparison between different methods for the detection of coronaviruses.

**Table 2:** Summary of presented methods for detection of coronaviruses

Technique	Sensitivity	Specificity	LOD	Cost	Detecting time	Advantages	Disadvantages
CT scan	Very high	Low	High	100–1000 USD	30 min	*Short executive time *High-resolution image *Providing unique medical information	*Exposure to the radiation *Expensive *No information about the cause of disease
Real-time PCR	10 copies of nucleic acid per µL	Moderate	Low	40–60 USD	4–6 h	*Popularity and credibility among scientists *Determining the exact infection stage *Detecting low level of RNA/DNA	*Not suitable for screening after clearance *Variety in the replication site of virus and sampling site *Showing only acute infection
LAMP	10–100-fold higher than RT-PCR	Moderate	Low	Low	< 1 h	*Low-cost equipment *No need for thermal alternations *Possibility to be reported with naked eyes	*Can be inhibited with some constituents within samples *Low versatility *Possibility of primer–primer interactions
ELISA	0.01–0.1 ng	High	Moderate	20–40 USD	1–2 h	*Detecting antigens at nano- or picogram level. *High detection throughput *Ease of performing *Quantitative measurement *Applicability with a variety of samples	* Temporary reading time *Low reported information
Sensing/Biosensing assays	1 pg–200 ng per mL	High	Very low	5–10 USD	Less than 10 min	*Short executive time *Detecting targets in very low amount *Cheap *Longer response time and high stability *No need for biocatalysts	*Need for sample preparation *Tedious process *Affected by temperature or pH

## Summary and Future Outlooks

In this paper, we describe some important detection methods of various types of coronavirus, including clinical and sensor-based methods. Currently, the lack of any rapid, available, and reliable POC detection method gives rise to the progression of COVID-19 as a horrible global problem. Most of the countries around the world had underestimated the novel coronavirus infection and ignored any plan to prevent the spread. Since other clinical detection methods such as ELISA or RT-PCR were feasible for previous epidemic viral infections, the SARS-CoV-2 was also accounted for the mild respiratory viral infection and assessed by the earlier methods. However, this new coronavirus has less mortality rate than SARS or MERS-CoV; SARS-CoV-2 is transmitted more quickly from human to human. Moreover, this virus can modify its genome via a mechanism called template switching that gives it the ability to change the virus RNA sequence and even in the amino acidic stage [171].

Immunoassay-based methods such as ELISA are among common methods to detect a variety of virus-derived antigens or their corresponding antibodies for diagnostic purposes or determining the efficiency of vaccination. The sensitivity of SARS-CoV N-based IgG ELISA is significantly higher than that of SARS-CoV S-based IgG ELISA, but the sensitivity of SARS-CoV-2 IgG/IgM still remains to be studied. Hampered results are among serious challenges and may occur due to a range of problems (false negativity, noise, unspecific reactions). Additionally, the method requires a relatively long time to be completed. In general, ELISA kits are costly and need skilled personnel to conduct the procedure, use the equipment, and interpret and report the results. These challenges warrant for developing other methods to overcome these issues. Immune-PCR (IPCR) is a method that uses both specificity of antibody–antigen and sensitivity of PCR. ELISA sensitivity is not enough to identify viral protein of low abundance, while it can detect any protein and PCR cannot be used directly for viral protein detection since it does not utilize antibodies. IPCR reproducibly increases sensitivity (10- to 1000-fold) for detection of pico–femtogram analyte from serum/urine, while provides multiplexing option because of ELISA and PCR combination (through an antibody–oligonucleotide conjugate) [172].

The delicate detection and enumeration of viruses are done by an excellent tool called real-time RT-PCR, where the improved product created through each cycle is quantified either by using SYBR Green or by numerous fluorescent probe chemistries to diagnose SARS-CoV-2; although RT-qPCR is specific, its false-negative rate cannot be overlooked because of the severe aftereffects of missed diagnosis. At present, next-generation sequencing (NGS) is progressively employed to understand the molecular epidemiology, transmission, and characterization of viruses. By performing a single test, large deposits of genes present in clinical samples can be identified rather than employing gene-by-gene analysis. In June 2020, FDA granted an EUA to Illumina, Inc. for the first COVID-19 diagnostic test utilizing NGS technology. It is the first authorization for NGS to use in diagnostics. In this test, 98-bp DNA fragment of SARC-COV-2 genome was used. Its limits are 1000 copy of viral genome per milliliter of sample, and it showed 97% specificity and 98% sensitivity. By using NGS test, not only virus can be detected but also it will provide sequence information of SARS-COV-2, which can be used to understand more about mutation and route of transmission over time [173].

Among the other approaches investigated between molecular approaches and PCR or recognition of viral diseases, LAMP-based methods are of great importance due to their numerous benefits. The most spectacular advantages of LAMP assays are the use of less equipment, availability, cheapness, quick detection, and also technically sound test. Efficient primer design is the main precondition of LAMP utilizing in a fertile way. As an accurate, fast, and cheap method for diagnosis, LAMP is employed to selectively amplify the target nucleic acid in isothermal conditions. There is no need for complex instruments; a water bath is enough and the completion time is about 1 h through LAMP method. In this method, the results are directly visible when SYBR Green or hydroxynaphthol blue (HNB) or calcein dyes are added. When a ladderlike outline is observed, we can also use gel electrophoresis. The outcome of improvements in molecular biology and biotechnology field is that the primer designing becomes a little tranquil, but there is a need for more investigations in the future for false positivity of the LAMP reaction.

As reactions can be done and outcomes can be read without opening reaction tubes, it demonstrates the great potential of LAMP in disease recognition. CT scan and RT-qPCR are notable for the diagnosis of SARS-CoV-2; most of the clinicians proposed that CT scans should be one necessary auxiliary diagnostic method; Table 2 summarizes the comparison between different methods for detection of coronaviruses. Because it is more sensitive than RT-qPCR for cases with a high clinical suspicion of the infection with negative RT-qPCR screening, a combination of repeated RT-qPCR tests and chest CT scan may be helpful.

To overcome these challenges, we need to design a novel detection method solving the problems of old clinical methods as well as the ability of conformation with the features of this new coronavirus. Immunosensor-based techniques are designed and used solely to eliminate the disadvantages of old clinical methods and are, therefore, of great importance. They have several benefits, including rapid detection, low cost, availability, and ability to detect the low concentrations of the desired material. These techniques are also appropriate to detect several parts of coronavirus particles, which can solve the problem of coronavirus mutations and false-negative results. On the other hand, in comparison with sensing, biosensing, apta-sensing, and immunosensing assays, LFAs (lateral flow assays) are more important and attractive POC devices for widespread uses. The important benefits they can provide are simple test processes, low sample volume requirements, rapid analysis, no necessity for expert staff, low cost of performance, user-friendly, and also cost-effective characteristics. Given the needs of communities for detecting the infectious agents at the early stage to inhibit the wide scale of prevalence, future studies must be aimed to find a proper method rather than usual clinical methods, as the rapid POC test for finding pathogenic viral infections and in particular coronavirus earlier than ever. The health care provider systems of all countries should be equipped with a distinguished platform having the ability to design new methods to detect likely future mutated viruses to prevent further epidemics or pandemics.

Development of point-of-care testing (POCT) of IgM/IgG (immune identification technology), biosensing assay and nanobiosensors for precise, suitable, and rapid detection of N protein biomarker of SARS-CoV, immunosensor array chip, microarray-based detection, LFA detection methods with higher sensitivity, all are ongoing endeavor. LFA with multiple functions, as well as biosensors and nanobiosensors tracers and accompanied detecting devices, may help to upgrade the efficacy of detection approaches. These biosensors are hoped to be utilized for detecting the SARS-COV2 virus as the accurate commercial biosensors that are now available in the market for diagnosis of HIV and influenza.

Some high-sensitivity biosensors are moving forward toward clinical trials. As example, one can mention chromatographic immunoassays for detecting influenza viruses (types A and B). These tests provide a colorimetric and qualitative identification of the virus in respiratory secretions. Other commercial biosensors for influenza A and B virus detection are as follows: Quidel, USA; Alere, USA; Directigen EZ Flu A + B, USA; SAS FluAlert A&B, USA; Coris BioConcept, Belgium, Thermo, USA; and OraSure Technologies, Inc., USA. There are also examples of these biosensors for HIV that are now available on the market: Runbio Biotech Co. Ltd., China; Alere, USA; Standard Diagnostics, Inc., Korea; and JAL Innovation, Taiwan [68, 174].

In the upcoming years, we should move toward producing cheap multifunctional biosensors that can support all the necessary detecting phases in a simplified manner. Also, we should solve the problem of complex storage requirements of the reagents, for example, by keeping all of them on a chip. The output of biosensors can be enhanced by applying biosensors with multiplexing features. Furthermore, the outputs should be presented quantitatively to obtain more accurate and more accessible (e.g., smartphone applications) results. Developing smaller size platforms is one approach toward applying such phone apps, as well as utilizing LFA, biosensors, and nanobiosensors detection techniques. Therefore, for developing portable detection systems which give the possibility for remote diagnostics, the size of detection systems should become as minimized as possible. Nevertheless, these miniaturized systems must be able to deliver all the functions of an optimal diagnostic test, such as simplicity, affordability, user-friendly, and the abilities to store, import, and export results.

Briefly, developing point-of-care biosensors and nanosensors with these features can provide the opportunity to rapidly screen the SARS-COV2 virus in populations and confine the virus spread.
